# A comparison of oral omeprazole and intravenous cimetidine in reducing complications of duodenal peptic ulcer

**DOI:** 10.1186/1471-230X-6-2

**Published:** 2006-01-11

**Authors:** Manouchehr Khoshbaten, Ebrahim Fattahi, Nosratollah Naderi, Farzaneh Khaleghian, Mohammadreza Rezailashkajani

**Affiliations:** 1 Liver and Gastrointestinal Diseases Research Center, Tabriz University of Medical Sciences, Tabriz, Iran; 2 Drug Applied Research Center (DARC), Tabriz University of Medical Sciences, Tabriz, Iran; 3 Research Center for Gastroenterology and Liver Diseases, Shaheed Beheshti University of Medical Sciences, Tehran, Iran; 4 Tabriz University of Medical Sciences, Tabriz, Iran

## Abstract

**Background:**

Gastrointestinal bleeding is a common problem and its most common etiology is peptic ulcer disease. Ulcer rebleeding is considered a perilous complication for patients. To reduce the rate of rebleeding and to fasten the improvement of patients' general conditions, most emergency departments in Iran use H2-blockers before endoscopic procedures (i.e. intravenous omeprazole is not available in Iran). The aim of this study was to compare therapeutic effects of oral omeprazole and intravenous cimetidine on reducing rebleeding rates, duration of hospitalization, and the need for blood transfusion in duodenal ulcer patients.

**Methods:**

In this clinical trial, 80 patients with upper gastrointestinal bleeding due to duodenal peptic ulcer and endoscopic evidence of rebleeding referring to emergency departments of Imam and Sina hospitals in Tabriz, Iran were randomly assigned to two equal groups; one was treated with intravenous cimetidine 800 mg per day and the other, with 40 mg oral omeprazole per day.

**Results:**

No statistically significant difference was found between cimetidine and omeprazole groups in regards to sex, age, alcohol consumption, cigarette smoking, NSAID consumption, endoscopic evidence of rebleeding, mean hemoglobin and mean BUN levels on admission, duration of hospitalization and the mean time of rebleeding. However, the need for blood transfusion was much lower in omeprazole than in cimetidine group (mean: 1.68 versus 3.58 units, respectively; p < 0.003). Moreover, rebleeding rate was significantly lower in omeprazole group (15%) than in cimetidine group (50%) (p < 0.001).

**Conclusion:**

This study demonstrated that oral omeprazole significantly excels intravenous cimetidine in reducing the need for blood transfusion and lowering rebleeding rates in patients with upper gastrointestinal bleeding. Though not statistically significant (p = 0.074), shorter periods of hospitalization were found for omeprazole group which merits consideration for cost minimization.

## Background

Gastrointestinal bleeding is a common problem in gastroenterology and its most common etiology is peptic ulcer disease which accounts for 50% of the cases with gastrointestinal bleeding [1]. In the majority of the cases, bleeding ceases spontaneously [2]. However, some factors may contribute to continuous bleeding and rebleeding which may aggravate prognosis. The risk factors for continuous bleeding or rebleeding include age over 60 years, gastric ulcer, severity of the first episode of bleeding, rebleeding in hospital, persistent or recurrent bleeding, and underlying diseases such as hypertension, chronic obstructive pulmonary disease, diabetes mellitus, recent myocardial infarction, cerebrovascular accidents and renal failure [3].

Some endoscopic findings, the so-called endoscopic evidence of rebleeding risk, can increase the risk of rebleeding up to 10% to 30% in peptic ulcer patients [4-6]. Endoscopic treatment of ulcers with higher risk of bleeding can decrease the rate of rebleeding, associated complications and even mortality [7-9]. H2-receptor blockers and proton pump inhibitors have been extensively used in the treatment of peptic ulcers. Omeprazole, a proton pump inhibitor, prevents rebleeding by increasing gastric pH and stabilizing blood clots in the base of the ulcer. Nevertheless, in several studies, H2-receptor blockers were not found to have any strict and persistent effect on gastrointestinal bleeding and did not decrease the rates of rebleeding, surgical interventions or mortality [6,10,11].

The role of treatment with proton pump inhibitors in the patients with active or recent ulcer bleeding is controversial. If given in an adequate dose by continuous intravenous infusion, proton pump inhibitors can maintain intragastric pH at 6 or above. At such levels of pH, peptic activity is minimized, platelet function is optimized, and fibrinolysis is inhibited; these effects may help to stabilize clot formation in an ulcer.

In case intravenous treatment is unavailable or particularly expensive, oral treatment would be appropriate. Furthermore, it would be less costly for any patient with recent ulcer bleeding who did not require endoscopic haemostatic therapy. One systematic review and meta-analysis found that proton pump inhibitors reduce the risk of ulcer rebleeding but does not influence overall mortality from ulcer bleeding [12].

Considering the absence of intravenous proton pump inhibitors in the conventional pharmacopoeia of Iran, intravenous cimetidine 200 mg is used every six hours before performing an upper gastrointestinal endoscopy for treating upper gastrointestinal bleeding in emergency departments in the majority of medical care centers in the country. It is also noteworthy that intravenous cimetidine is more available than intravenous ranitidine in Iran. The aim of this study was to compare the therapeutic effects of oral omeprazole and intravenous cimetidine (immediately started in the emergency department) on decreasing rebleeding rates, duration of hospitalization and the need for blood transfusion. We also tried to adhere to CONSORT statement  in reporting this clinical trial.

## Methods

All of the patients over 12 years of age with upper gastrointestinal bleeding (hematemesis and/or melena) referring to emergency departments of Imam and Sina hospitals in Tabriz, Iran were assessed for inclusion criteria of the study. The patients with gastrointestinal bleeding due to duodenal ulcer and endoscopic risk factors for rebleeding were candidates for inclusion in the study. Endoscopic risk factors for ulcer rebleeding were hematin-covered flat spots, sentinel clot, visible vessels, arterial oozing bleeding, and arterial spurting bleeding. Exclusion criteria were gastrointestinal bleeding not caused by duodenal ulcer, bleeding caused by drugs (e.g. anticoagulants) except NSAIDS or underlying diseases (e.g. thrombocytopenia and coagulopathies), and absence of endoscopic risk factors of rebleeding. A computerized random-number generator was used to produce the random allocation sequence on an equal basis (probability of receiving each treatment was set to be 50% before assignment). Concealment of allocation was performed using sequentially numbered, sealed, opaque envelopes. A written informed consent was obtained from each patient before allocation.

All of the patients with upper gastrointestinal bleeding were treated with intravenous cimetidine (Darou-Pakhsh, Iran) 200 mg every 6 hours and underwent upper gastrointestinal endoscopy within 12 to 24 hours of hemodynamic stabilization and gastric lavage. In cases with hemodynamic instability or continuous bleeding, endoscopy was performed on an emergency basis and those with endoscopic risk factors for rebleeding were treated with alcohol (50% solution) and adrenaline (1/10000 solution). Thermal coagulation was not used because it was not available in our center.

Patients meeting inclusion criteria were randomly assigned (as explained above) to two groups of equal number. One group was treated with continuous intravenous cimetidine (Darou-Pakhsh, Iran) 200 mg every six hours for 3 days followed by oral cimetidine (Darou-Pakhsh, Iran) 400 mg every 12 hours to the 14^th ^day after admission. In the other group, intravenous cimetidine was discontinued and omeprazole suspension (the contents of one 20-mg omeprazole capsule [Exir, Iran] in 50 ml of normal saline) was prescribed orally every 12 hours for 3 days and then was replaced by oral omeprazole 20-mg capsules every 12 hours to the 14^th ^day after admission. Omeprazole administration in form of suspension was because the patients in emergency department had an NG-tube and could not take omeprazole capsules early in hospitalization. Patients were kept blind to the two intervention modes however it was not possible to keep health care providers blind to interventions since the form of administered drugs (oral cimetidine tablets and omeprazole suspension) was apparently distinguishable early in treatment.

Active bleeding was defined as observation of bright-red blood on a gastric lavage. The duration of active bleeding was defined as the period of time between the first and the last gastric lavage showing bright-red blood on the frequent lavages routinely performed as standard treatment for patients with upper gastrointestinal bleeding in the emergency department. Rebleeding was defined as observation of red blood in the stomach, a drop of serum hemoglobin more than 2 gr/dl during 24 hours, continuous melena for more than 7 days, or instability of vital signs (defined as a pulse rate more than 110 per minute, positive tilt sign, or a drop of systolic blood pressure below 90 mmHg in supine position).

In the cases with rebleeding; the time interval from primary bleeding, number of rebleeding periods, and methods of treatment were recorded. The results were analyzed with SPSS software version 11.5 using chi-square and *t *tests. *P *values less than 0.05 were regarded statistically significant.

## Results

**Figure 1:**
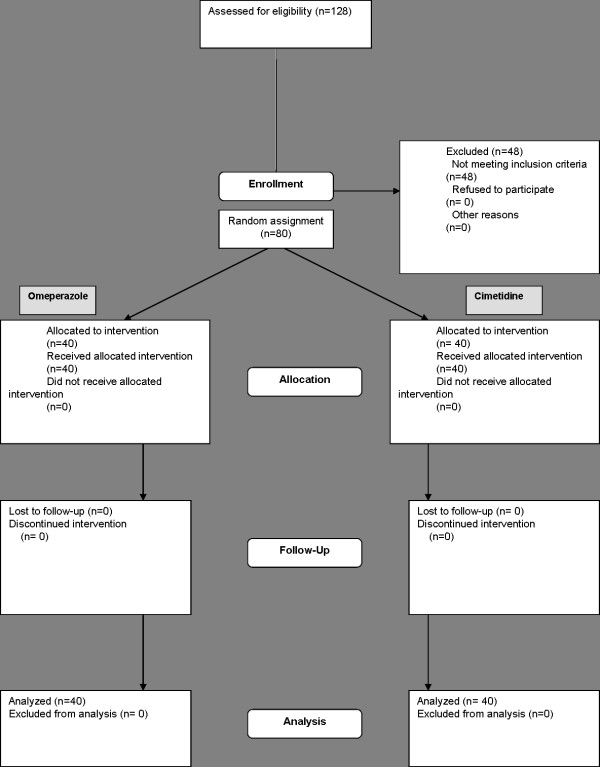
CONSORT flow chart of the clinical trial comparing oral omeprazole and intravenous cimetidine in reducing complications of duodenal peptic ulcer in 80 Iranian patients.

Of 128 patients with upper gastrointestinal bleeding (hematemesis or melena) referring to emergency departments of Imam and Sina hospitals in Tabriz, 80 met the inclusion criteria of the study. Figure 1 shows the CONSORT diagram showing flow of cases in this clinical trial. The mean age (± SD) of all patients was 51.53 (± 18.1) years. The mean age was 53.5 and 49.5 years for cimetidine and omeprazole groups, respectively. Statistical analysis interestingly showed that case and control groups were matched for sex (p = 0.639) and age (p = 0.332). There was no statistically significant difference between cimetidine and omeprazole groups in regards to sex, age, alcohol consumption, cigarette smoking, NSAID consumption, endoscopic risk factors for rebleeding, mean hemoglobin or mean BUN levels on admission, duration of hospitalization and mean time of rebleeding (Tables 1 and 2). It is noteworthy that though age and sex matching was not aimed from the start of the study, the case and control groups were incidentally found to be age and sex matched at the time of statistical analysis.

The mean duration of active bleeding (see methods) was longer in omeprazole group compared with cimetidine group. Rebleeding rate in omeprazole group (15%) was significantly lower than that of cimetidine group (50%) (p < 0.001). Surgical interventions were needed for 6 cases in cimetidine, and only 1 case in omeprazole group. While three patients in cimetidine group died (two because of rebleeding and one due to cerebrovascular accident), there was only one dead case in omeprazole group (because of pancreatic malignancy).

## Discussion

Many studies have confirmed that Asian patients with duodenal ulcer had smaller maximal acid output (MAO) than Caucasian patients. [13] Khuroo et al also demonstrated that the parietal cell mass in duodenal ulcers was much lower in Indian patients than in those in Western countries [14]. This evidence may partly justify considering using oral (versus intravenous high-dose) omeprazole in controlling peptic ulcer bleeding in patients of this study.

Ulcer healing time is shorter with omeprazole compared with that with cimetidine or ranitidine [15]. Since pepsin is activated in pH levels lower than 4 causing clot lysis [10] and omeprazole increases gastric pH, it can stabilize the blood clot in ulcer base and so prevents rebleeding [16,17]. Lau et al used adrenaline injection and thermal probe for bleeding control and omeprazole infusion for rebleeding prevention and found a rebleeding rate of 6.7% (mortality rate: 4.2%) in omeprazole and 22.5% (mortality rate: 10%) in placebo group [18]. Schaffalitzky et al used intravenous omeprazole with and without thermal coagulation or electrocoagulation and found the first-72-hour improvement rate to be 94% in omeprazole, and 79.7% in placebo group. Mortality rates in their study were 7.7% in cimetidine and 8.5% in omeprazole group [19]. In our study, rebleeding rate was 50% in cimetidine and 15% in oral omeprazole group which showed a statistically significant difference (p = 0.001) and confirmed the similar results from previous studies [44, [5,20]]. While the rebleeding rate in cimetidine group was 3 times higher than that in omeprazole group in our study, Lin et al found rebleeding rates to be 6 times higher in cimetidine group [4]. Overall, 25% of our patients experienced rebleeding in rates similar to those found by previous studies (10% to 30%) including the study of Katschinski (11%) [21,22]. In all cases, rebleeding occurred in patients with endoscopic risk factors for rebleeding.

In Lin et al study, the mean duration of hospitalization was 6 days in cimetidine and 7 days in omeprazole group which was not significantly different (p > 0.05) [4]. Similarly, the difference in duration of hospitalization between two groups in our study was not statistically significant (p = 0.074) though Kaviani et al found this difference to be significant (p = 0.0032) [23].

In our study, the mean amount of blood transfusion was 3.58 units for cimetidine and 1.68 units for omeprazole group which showed a statistically significant difference (p = 0.003); a result previously found by Lin et al (p = 0.008) [4].

Mortality in cimetidine and omeprazole groups was 2% versus none in Lin et al study (p > 0.05)^4 ^and 1.99% versus 1.84% in the study by Li et al (p > 0.05)^13^, respectively. In our study, mortality rate was 7.5% in cimetidine and 2.5% in omeprazole group; we similarly found no significant difference (p = 0.24). The total mortality rate in two groups in our study was 10% while the same figure was 8.5% in Katschinsky and 6% to 7% in Barkun et al studies [11,21].

All death cases in our study were among the patients older than 60 years of age. Our results were similar to Katschinski's findings [21] but in conflict with the findings of Segal et al [24]. Furthermore, the majority of death cases in our study were among patients with underlying diseases though rebleeding was the cause of 2 deaths in cimetidine group. This result was different from that of Katschinski's study [21]. There was only one death because of pancreatic malignancy in omeprazole group.

## Conclusion

In conclusion, this study suggested that patients treated with oral omeprazole had lower rebleeding rates (p = 0.001) and lower need for blood transfusion (p = 0.003) compared with those treated with intravenous cimetidine.

## Competing interests

The author(s) declare that they have no competing interests.

## Authors' contributions

MK: Proposed the main idea of research, supervised the project and participated in project design, data analysis, and manuscript writing.

EF: Participated in design of research, data analysis and coordination of project in clinical setting.

NN: Participated in manuscript writing, data analysis and revising manuscript.

FK: Participated in data collection and analysis.

MR: Performed manuscript revisions and language edit, made manuscript CONSORT compatible and wrote response to reviewers.

## Pre-publication history

The pre-publication history for this paper can be accessed here:



## Figures and Tables

**Table 1:** The characteristics of 80 patients with upper gastrointestinal bleeding treated with intravenous cimetidine or oral omeprazole.

	Cimetidine group No (%)	Omeprazole group No (%)	p values
Hx of alcohol	4 (10)	7 (17.5)	0.33
Hx of cigarette smoking	14 (35)	15 (37.5)	0.816
Hx of NSAIDs	19 (47.5)	18 (45)	0.823
Hx of epigastric pain	22 (55)	21 (52.5)	0.823
Pre-existing disease	18 (45)	17 (42.5)	0.283
Hx of gastrointestinal bleeding	3 (7.5)	10 (25)	0.034
Mean Hb level (gr/dl)	9.53	10.04	0.392
Mean BUN (mg/dl)	16.55	10.72	0.629
Blood transfusion (units)	3.58	1.68	0.003
Mean interval of rebleeding (days)	2.1	2.8	0.34
Rebleeding	20 (50)	6 (15)	0.001
Duration of hospitalization (days)	7.46	5.6	0.074
Mortality	3 (7.5)	1 (2.5)	0.24

Hx: History

**Table 2:** The frequency of endoscopic findings according to Forrest classification in 80 patients with upper gastrointestinal bleeding treated with intravenous cimetidine or oral omeprazole. P values for all classes were 0.9.

*Endoscopic findings*	*Cimetidine group No (%)*	**Omeprazole *g*roup No (%)**
Arterial spurting bleeding (IA)	3 (7.5)	2 (5)
Arterial oozing bleeding (IB)	15 (37.5)	13 (32.5)
Visible vessel (IIA)	5 (12.5)	3 (7.5)
Sentinel clot (IIB)	12 (30)	15 (37.5)
Hematin covered flat spot (IIC)	5 (12.5)	7 (17.5)
